# Piloting electronic screening forms in primary care: findings from a mixed methods study to identify patients eligible for low dose CT lung cancer screening

**DOI:** 10.1186/s12875-017-0666-5

**Published:** 2017-11-28

**Authors:** Mary Ann O’Brien, Frank Sullivan, Andrea Carson, Rabiya Siddiqui, Saddaf Syed, Lawrence Paszat

**Affiliations:** 1 Department of Family and Community Medicine, University of Toronto, 500 University Avenue, Fifth Floor, Toronto, ON M5G 1V7 Canada; 2 North York General Hospital, Toronto, ON Canada; 3 Medical School, University of St Andrews, Scotland, UK; 4 Sunnybrook Research Institute, Toronto, ON Canada; 5 Institute for Clinical Evaluative Sciences, Toronto, ON Canada

**Keywords:** Lung cancer screening, Primary care, Health services research, Low dose computed tomography (LDCT)

## Abstract

**Background:**

Recent evidence suggests that screening with low dose computed tomography (LDCT) scans significantly reduces mortality from lung cancer. However, optimal methods to identify potentially eligible patients in primary care are not known. Using brief electronic screening forms administered prior to a primary care visit is a strategy to identify high risk, asymptomatic patients eligible for LDCT screening. The objective of this study was to compare the acceptability and feasibility of using brief electronic versus paper screening forms to identify eligible patients at high risk of developing lung cancer in primary care.

**Methods:**

A mixed method pilot comparative study was conducted in primary care. Practices were allocated to an electronic form (e-form) group or a paper-based form (p-form) group. Allocation was randomly assigned for the first practice then by alternation. Patients in the e-form practices completed forms at home via the web or in the waiting room on a tablet. Patients in p-form practices completed forms in waiting rooms. Interviews were conducted with patients, administrators, and primary care physicians (PCPs) about their experiences.

**Results:**

Six of 30 (20%) eligible practices agreed to participate. Over the 16-week study period, a total of 831 of an expected 1442 patients (58%) aged 55–74 years were enrolled; 573/690 (83%) patients in the e-form group and 258/752 (34%) in the p-form group. Of the 573 participants in the e-form group, 335 (58%) completed forms via the web; 238 (29%) did so via tablet. Twenty-four interviews were conducted with 15 patients, 5 administrative staff and 4 PCPs. Patients were willing to discuss lung cancer screening eligibility with their PCP. Staff members expressed low administrative burden except for an extra step to link appointment information to patient demographics to identify eligible patients. PCPs indicated that forms were reminders to discuss smoking cessation. PCPs in the e-form group reported that patients asked questions about screening.

**Conclusion:**

There was fairly low uptake by primary care practices. For e-forms to be feasible in practice workflow, electronic medical record software needs to link appointment information with patient eligibility requirements. The use of brief pre-consultation electronic screening forms for LDCT eligibility encouraged PCPs to discuss smoking cessation with patients.

## Background

The National Lung Screening Trial in the United States and the Lung Cancer Screening Trial in the United Kingdom have demonstrated that screening with low dose computed tomography (LDCT) scans significantly reduces mortality from lung cancer [1, 2]. Optimal methods to identify potentially eligible asymptomatic patients in primary care are not known, although web-based technologies may be useful. For example, software is now available which allows patients attending primary care appointments to receive and respond to electronic questionnaires before their consultation. Responses to electronic questionnaires can then be used for different purposes such as determining eligibility for clinical studies, populating the electronic medical record (EMR) and providing an opportunity for ‘just-in-time’ counseling by primary care providers (PCPs). For example, in New Zealand, researchers developed an electronic tool for primary care called eCHAT (electronic case-finding and help assessment tool) [3]. The tool was used by patients in the waiting room prior to a visit or via the Internet in the community. The goal of the tool was to encourage active participation by patients in decision-making and self-management practices.

In the context of LDCT lung cancer screening, pre-consultation software could be used to facilitate identification of potentially eligible patients. This software may also help to remedy physician inconsistencies in recommending high-risk patients to LDCT that was identified as problematic in a previous study [4], by contributing to a more formalized screening process. Information from pre-consultation screening forms may help PCPs to counsel their patients about their risk for lung cancer and the potential benefits and risks of LDCT lung cancer screening, and refer appropriate patients to a screening program.

Identifying and describing the potential impact of electronic technologies on health care is necessary to better prepare for the potential myriad of challenges that come with implementing such technologies in primary care [5]. The goal of the current study was to assess two pre-screening strategies to identify eligible patients for LDCT should a lung cancer screening program become available in Ontario. At the time of this study, an organized LDCT screening program for lung cancer was not available in Ontario although pilot work for a potential program is underway (https://www.cancercareontario.ca/en/guidelines-advice/cancer-continuum/screening/lung-cancer-screening-pilot-people-at-high-risk).

The design and implementation of this study were based on our views of how the primary care clinical process would unfold. The pre-screening form used in this study was not intended to determine eligibility for LDCT screening; instead, it was intended to identify patients who were at high risk for lung cancer, who could then potentially be referred to a more detailed screening assessment and possible LDCT screening. This paper reports the acceptability and feasibility of electronic and paper-based strategies to make a preliminary assessment of lung cancer risk in asymptomatic patients in primary care.

## Methods

### Pilot comparative study (quantitative component)

#### Participants

Family physicians affiliated with a single community hospital in Ontario were identified using the College of Physicians and Surgeons of Ontario database (http://www.cpso.on.ca/Public-Information-Services/Find-a-Doctor/). The research team identified and contacted 30 physicians from this list; 6 physicians representing 6 practices agreed to participate. Patients were eligible to participate if they were between 55 and 74 years (y) and had a scheduled appointment with a clinician in one of the six practices that enrolled in the study. Based on the number of patients aged 55–74 y seen in 2015 in each practice and given a study period of 16 weeks, we estimated that about 1442 patients would be eligible during the study period.

#### Design

**Fig. 1:**
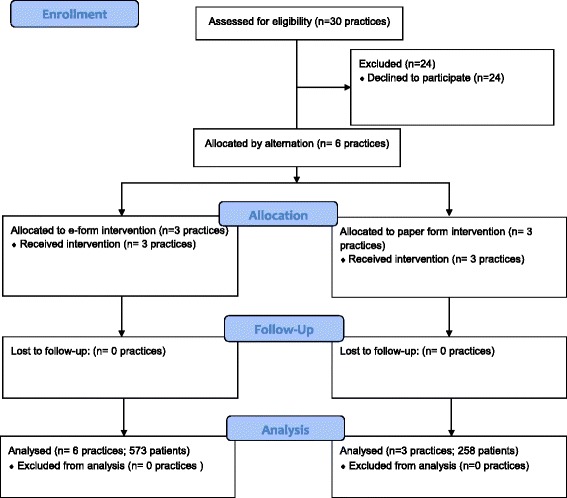
Study Flow

Six practices were assigned to either an electronic form (e-form) group (completion of screening forms via pre-consultation software) or a paper form (p-form) group (completion of paper forms in the waiting room). To avoid unconscious selection bias, the first consenting practice was assigned by coin toss; thereafter, practices were assigned to groups by alternation. All six practices completed the study (Fig. 1).

#### Data collection

All patients completed a questionnaire that included three questions pertaining to lung cancer screening (Table 1), demographics, and Internet preferences; administrative staff and physicians completed a demographic form. Information regarding primary practice setting, type of practice, and practice fee structure was obtained from physicians at four out of six practices. For the remaining two practices, information was obtained using the websites of the College of Physicians and Surgeons in Ontario and the North York Family Health Team. To estimate the number of potentially eligible patients in each practice over the 16-week study period, we used the Canadian Primary Care Sentinel Surveillance Network (CPCSSN) database for five of six practices. We obtained the actual number of eligible patients aged 55–74 y seen in 2015 and prorated this value to 16 weeks. For the sixth practice, we imputed the number of eligible patients seen in one year based upon the average of the actual number of eligible patients divided by the practice size for the other practices and multiplied by the practice size of 2000 patients.

**Table 1:** Pre- Screening Questions

The three questions below were included on the lung cancer screening form. The response options were “yes” or “no”. 1. Are you between the ages of 55 and 74? 2. Have you smoked cigarettes for at least 20 years in your lifetime? 3. Have you usually smoked 10 or more cigarettes per day?	

#### Interventions

Two pre-consultation strategies were compared. The intent of a pre-consultation strategy was that physicians would receive just-in-time information about the patient’s responses on the pre-screening form and would discuss lung cancer screening or smoking cessation in the visit. In the e-form group, pre-screening forms were sent electronically to eligible patients to complete at home via the Internet. This strategy was intended to place minimal demands on administrative staff. If the EMR indicated that the form was not completed, patients were asked to complete the form on a tablet in the waiting room prior to their visit. The e-form strategy was pilot-tested with 12 patients in one practice. In the p-form group, eligible patients were asked to complete a paper version in the waiting room. Brief descriptions of the implementation of each strategy are provided below.

#### E-form practices

The implementation of the e-form varied depended on each practice’s EMR capability and compatibility with Oceanwave software (CognisantMD http://www.cognisantmd.com/). In two of three practices, the following procedure was used: At the beginning of the week, all eligible patients with a scheduled appointment and with a cell phone number or email address on file were sent a study invitation by email or text. Patients were then sent a url link via the Oceanwave software to a web version of the lung cancer screening form, as well as a demographic and Internet preferences questionnaire**.** When patients checked in for their appointment, the EMR would indicate if the screening form was complete. Patients who had not yet completed the form were invited to participate by clinic staff and given a tablet on which to complete the form in the waiting room prior to their appointment. Data from the tablet were uploaded to the EMR. The third practice lacked EMR and Oceanwave software compatibility, and had few patient emails and cell phone numbers on file. Eligible patients at this clinic were approached by a practice research facilitator to complete the screening form via tablet in the waiting room.

#### P-form practices

The implementation process at p-form practices varied and depended on administrative staff preference and daily office functioning. Each practice displayed study information on posters in the waiting room. In one practice, the receptionist provided forms to eligible patients. If they agreed to participate, patients were asked to return completed forms to the receptionist or to a collection box in the waiting area. In the remaining two practices, staff waited for interested patients to approach them. However, staff at one of these two clinics reported low participation and thereafter actively invited patients to the study.

### Outcomes

The primary study outcome (acceptability) was the proportion of practices who agreed to participate of those approached. The secondary outcomes (feasibility) were: 1) the proportion of patients with completed forms in each group; 2) the proportion of patients in the e-form group who completed the forms via the Internet versus a tablet. Acceptability of completing pre-consultation forms was also explored during interviews with patients, administrative staff, and physicians.

### Analysis

Data were analyzed descriptively using counts and proportions.

### Qualitative study component

#### Patients

After completing the screening form, patients in both e-form and p-form groups were invited to participate in a brief semi-structured telephone interview about their experience with the form. A purposeful sampling strategy was used to select the patients for interviews [6]. Two or three patients from each clinic who had completed the forms were contacted. A mix of patients at high risk, low risk (as determined by their responses to the smoking questions), and who indicated both low and high comfort using technology were contacted for an interview. Patients who completed a telephone interview were mailed a $25.00 gift card.

In the interviews, patients were asked about their overall experience completing the lung cancer pre-screening form, whether or not they had difficulties, if they would be willing to answer follow-up questions based on the form, and their general views on having lung cancer screening forms in primary care.

#### Administrative staff and physicians

Staff members were asked about their experiences implementing the screening forms, the impact on clinical functioning, and their interactions with patients. Physicians were asked about their overall impressions of the clinical process of having screening forms in their offices, and whether this had an impact on the subsequent clinic visit.

### Analysis

Two research team members analyzed the interview transcripts using an editing style of coding [7] using data management and analysis software (NVivo 9, QSR International). Three interviews were coded and results were compared until team members reached an agreement on the preliminary codes. The interview guide was revised throughout the analytic process as new codes were identified. Qualitative analytic techniques were used to inductively identify the main themes [8–10]. An audit trail including interview summaries and memos were used to document all major decisions taken during the study [11].

## Results

### Quantitative study results

#### Demographics

Estimates for the number of patients in each practice and the number of eligible patients are provided in Table 2. The estimated practice size varied from approximately 2000 to 7400 patients in the e-form group and 5000 to13500 patients in the p-form group. The estimated number of eligible patients in the practices ranged from 1400 to 1600 in the e-form group and 1100 to 2000 in the p-form group. All six practices were in a community-based setting. Five of six practices were part of a family health team. All six practices were structured based on an alternative payment plan.

**Table 2:** Description of primary care practices by pre-screening strategy to identify potentially eligible patients for lung cancer screening

	Electronic Form			Paper Form		
	Practice 1	Practice 3	Practice 4	Practice 2	Practice 5	Practice 6
Estimated practice size	7400	2000	5700	13,500	8100	5000
Estimated number of eligible patients	1600	Not known^a^	1400	2000	1700	1100
Actual number of eligible patients seen per year (2015)	1052	242^b^	948	1044	811	589
Number of physicians per practice	5	1	3	6	5	4

^a^Estimated number of eligible patients was not available from this practice

^b^Actual number of eligible patients seen in 2015 was not available from this practice. The number was imputed from the average of the actual number of eligible patients divided by the practice size for the other practices and multiplied by the practice size of 2000 patients

Across all practices in the 16-week study period, 831 of an expected 1442 patients (58%) aged 55–74 y were enrolled with 573/690 (83%) patients in the e-form group and 258/752 (34%) in the p-form group.

Table 3 describes patient participation by practice and by intervention group. The number of patients who would be potentially eligible for LDCT screening based on their smoking history as assessed by patient responses was 116/831 (14%) overall with 74/573 (13%) in the e-form group and 42/258 (16%) in the p-form group (Table 3).

**Table 3:** Patient recruitment by practice and pre-screening strategy (*n* = 831 patients)

	Electronic Form (573 patients)				Paper Form (258 patients)				
	Practice 1	Practice 3	Practice 4	Total	Practice 2	Practice 5	Practice 6	Total	Overall Total
Number of patients	361	14	198	573	54	193	11	258	831
Email link (at home)	235	0	100	335	N/A	N/A	N/A	N/A	N/A
Tablet (in waiting room)	126	14	98	238	N/A	N/A	N/A	N/A	N/A
Sex									
Male	146	10	52	208	20	69	3	92	300
Female	166	4	104	274	33	112	7	152	426
Lung Cancer Risk Category									
High Risk^a^	47	4	23	74	13	27	2	42	116
Low - Moderate Risk^b^	61	2	34	97	7	21	0	28	125
Low Risk^c^	250	8	141	399	32	133	6	171	570

^a^Represents patients who answered Yes (Y) to all three screening questions

^b^Represents patients who answered Y to age range and Y to one of the other two screening questions

^c^Represents patients who are within the eligible age range and answered No (N) to the second and third screening questions

#### Internet preferences

Results from the Internet preferences form (data not shown) indicated that 75% of participants were comfortable or very comfortable using computers and 80% used the Internet 1–7 h per day. Almost 80% of participants were comfortable using email and 56% were comfortable using smartphones. Almost 90% of participants indicated that they had a computer at home as well as Internet access.

### Outcomes

Primary outcome (acceptability): Six of 30 (20%) practices agreed to participate.

Secondary outcomes (feasibility):

1. Proportion of patients with completed forms in each group: In the e-form group, 68% of patients completed all questions as did 78% in the p-form group. Of the three questions related to lung cancer risk, 99% and 93% of patients in the e-form and p-form practices respectively, completed all three questions.
2. Proportion of patients in the e-form group who completed the forms via the Internet versus a tablet: Of the 573 patients in the e-form group, 335 (58%) completed the forms at home via the web and 238 (42%) completed the forms in the waiting room via tablet.

### Implementation of pre consultation screening strategies

During implementation of the electronic screening strategy, we found that the Oceanwave was not as compatible with EMR systems as we had anticipated despite pre-testing. In addition, the EMR capability for searching and identifying eligible patients was not as expected. For all e-form practices, we assumed that we would be able to identify patients within the specified age range who had an upcoming appointment. We assumed that all eligible patients would be sent the information and web link to the forms in a batch. However, this was not possible. The appointment search and email invitation had to be done manually each week by the front desk staff by searching the practice’s appointment schedule for patients who fit the age range and sending them email invitations for the study a few days before their scheduled visit.

### Qualitative results

Twenty-four interviews were conducted with 15 patients, 5 administrative staff and 4 PCPs. Of the 15 patients, three completed the form via the web, three completed the tablet version, and 8 completed the paper version. One patient could not recall the version that they had completed. Although we pre-specified that patients be able to read and understand English, one patient preferred to complete the telephone interview with the assistance of a translator. Of the 15 patients interviewed, 10 were women and 5 were men. All patients were between the ages 55 and 74 y. Of the 15 patients, 7 indicated that they smoked for a period of more than 20 y, and 5 indicated that during that time they had smoked more than 10 cigarettes per day.

Of the 5 administrative staff members, four were from p-form practices and one staff member was from an e-form practice and had experience with both web and tablet versions. All 5 administrative staff members were female; mean age was 35 y. Of the four physicians, two were from e-form practices and the other two were from p-form practices. All four physicians were male; mean age was 50 y.

### Theme 1: Patients found screening forms easy to complete

Patients expressed little to no difficulty completing the lung cancer pre-consultation screening form. For example, patients described their experience with the form as “fast”, “easy”, “efficient”, and “nothing to it”. All patients said that they would be willing to complete these types of screening forms prior to their primary care visit if they were implemented into practice. All patients were willing to answer follow-up questions and to discuss lung cancer screening eligibility with their PCPs.

### Theme 2: Screening forms caused minimal administrative burden for staff members

Administrative staff members expressed little administrative burden as a result of having these forms at their offices. The extra time that was required to participate in the study was primarily related to identification of eligible patients who had an appointment and either (i) making a list of patients to approach at the practice (p-form) or (ii) sending the survey via email (e-form). For example, in a p-form practice, an administrator noted:

“*Our office manager would go through the schedule like maybe at the beginning of the week, and any patients that fell within that age category … she would put a ‘Q’ [questionnaire] in the schedule. So anytime I saw the ‘Q’…I was prompted to hand out a questionnaire…As long as the office manager was there to do it, then it wasn’t an issue. [But] she wasn’t always able to add the questionnaires, so sometimes we would miss it if we weren’t paying attention while we were checking them in*.” (Admin 5)

In a practice that used both web and tablet versions, an administrator explained:

“W*e kind of developed on our own…system of … going through all our appointments a few days in advance and seeing who was eligible, emailing them, and then the day of, every morning looking through all the ones that we had e-mailed to see if they had responded yet, so then we knew exactly who we had to ask so that ended up working out very well*.” (Admin 3)

One practice described more success implementing the web versus tablet version of the form because of the nature of their office work flow.

“*We had a lot more success reaching [patients] by email I believe than tablet. Because of the nature of our office, we tend to get patients into rooms very quickly, and so, a lot of time, we didn’t have time to get them the tablet before the nurse was calling them in… The whole process, I think, was very positive. We did get a lot of responses. Just the tablet part didn’t work very well for us*” (Admin 3).

### Theme 3: Screening forms served as a reminder for physicians to discuss smoking cessation with patients

While the screening forms did not necessarily provide new information to physicians, they agreed that forms acted as a reminder to discuss smoking cessation and served as a reaffirmation to patients that smoking is harmful. For example: “*We’ve been working on smoking cessation for so long…so we’ve been incorporating it for a long time. So it’s hard to know about the degree to which this has an incremental impact on top of that. It’s certainly yet another reminder*” (Physician 1). Additionally: “*[The survey] would allow me to say that screening is not yet widely available but they would be an appropriate candidate if they met the criteria. So I think it got the discussion started*” (Physician 2).

## Discussion

The aim of this study was to assess the acceptability and feasibility of pre-consultation electronic screening forms to identify high-risk patients eligible for LDCT lung cancer screening. We targeted patients with a scheduled appointment only so that information would be ‘just-in-time’ and would be fresh in the minds of both patients and physicians [12]. We hypothesized that providing information just prior to the consultation could potentially facilitate a discussion about lung cancer screening and/or smoking cessation between physician and patient. However, several of the interviewed PCPs expressed that they would have preferred to target all eligible patients at their practices and not just those with a scheduled appointment.

The current study had a fairly low acceptance rate by practices of 20%. We do not know if low acceptance was related to lack of enthusiasm toward the pre consultation screening forms specifically on behalf of patients or practice administrative staff or lack of interest in participating in a research study. However, both patients and physicians who agreed to be interviewed expressed positive views about the electronic screening forms. With respect to feasibility, we found a high rate of completion of the three questions related to lung cancer risk. We were also interested whether the web version would be feasible as we had hypothesized that there would be less administrative burden as the EMR system could query the upcoming appointments list and automatically send invitations to eligible patients. Of the 573 patients in the e-form group, 63% completed the forms via the web link and 42% completed the forms via a tablet in the waiting room. We believe that this result can be attributed to technical difficulties with the EMR and/or the availability of cell phone numbers and email addresses in the practices. These problems could be resolved in the future with advances in EMR technology and improvements in capturing cell phone numbers and email addresses. In the current study, approximately, 88% of patients reported having Internet access at home and patients may be willing to provide email addresses to their primary care physicians.

Other studies have assessed the feasibility of introducing various computer-based software programs into primary care. For instance, Voncken-Brewster et al. assessed the feasibility of introducing a web-based self-management application into primary care in the Netherlands for patients living with chronic obstructive pulmonary disease. The authors argued that integrating this particular application in primary care could benefit patients and the organization of care. However, the authors found that patient interest in the application was not sustained through multiple revisits to the application [13]. Fleisher et al. assessed a web-based intervention to improve colorectal cancer screening in the United States (average risk and noncompliant to current screening recommendations). Using a randomized controlled trial design, they compared Internet versus print format of the intervention, and implicitly assumed that participants would embrace the web format. However, the authors found that the web intervention was underused in a “real world” setting [14]. It is possible that the lack of engagement in this web-based intervention was due to the unique password given to each participant that was required to log in to the program each time, and dwindling interest in the intervention. In the current study, patient completion of the three screening questions was high. This could be due to the brief nature of the form, as well the one-time only form completion. The frequency of administration of such screening forms in day-to-day practice is uncertain. Once a patient is referred to a lung cancer screening program, it is anticipated that the program itself would be responsible for follow up of patients.

The current study also reveals important insights regarding the ‘real world’ application of web-based software in primary care. Using normalization process theory, Mair et al. (2012) argue that the e-health literature has largely focused on organizational issues related to implementation, and neglected consideration of the wider social framework when introducing such technologies into practice [15]. The Normalization Process Model is a theoretical model that can be used to help conceptualize and understand the implementation of complex interventions in primary care. It raises such questions as: How effectively are practices able to organize and carry out a complex intervention in conjunction with their day-to-day activities? How does a complex intervention integrate into the organization in which it is set? [16]. Mair et al. (2012) suggest more empirical investigation is needed to examine how these e-services will affect the clinical interaction, performance, and work allocation, as well as different ways of engaging with professionals prior to and during their implementation [15]. In relation to the current study, by piloting an electronic pre-screening form (or “e-health intervention”) prior to the availability of LDCT screening, we gained important insights into the ways that this particular electronic strategy was implemented in practices, its perceived effect on practice functioning, and the willingness of patients to complete screening forms.

### Limitations

There are five limitations of this pilot study. First, we were unable to determine the actual number of eligible patients in each practice during the study period. Estimates of eligibility were based on the actual number of eligible patients seen in each practice in one year prorated to the 16-week study period. We do not know if the estimate we calculated was an over or under estimate of the actual number of patients seen in each practice.

The inability to obtain actual numbers of eligible patients during the study period was related to difficulties in linking the patients’ demographic information to the list of upcoming appointments. Improvements in EMR software capabilities will be needed to reduce barriers to the implementation of this type of pre-consultation screening strategy in primary care.

Second, this pilot study was conducted in six primary care practices affiliated with one community hospital. We do not know if our findings would be similar in other types of practice settings. Third, in the quantitative component of study, the first practice was assigned by coin toss and subsequent practices were assigned by alternation. The results of our study need to be interpreted with these design limitations in mind. In the future, a stronger design is warranted by including practices affiliated with more than one hospital and with random allocation to groups.

Fourth, patient recall during the qualitative interviews was limited. Although we attempted to avoid recall issues by selecting patients who had completed the survey within the previous three weeks, this was not always possible. The median number of days between form completion and interview was 70 days. Delays in contacting patients occurred because of non-responses to phone calls, incorrect numbers, and waiting for written consent forms to be returned by post rather than accepting verbal consent by phone. In the future, a shorter time span between form completion and interviews would be preferable.

Fifth, in this study, all forms were written and completed in English. Although one interview was conducted with the assistance of a translator, it is possible that we missed patients who may not have been able to read the pre-screening form, and who may have felt uncomfortable participating in an interview. Pre-screening forms that are distributed only in English are not able to capture data from diverse linguistic populations. Translated versions of the pre-screening form should be made available if a screening program were to be implemented in primary care in Ontario.

## Conclusions

Brief pre-consultation electronic forms for LDCT screening eligibility completed at home via the web, or on tablets in the waiting room were acceptable and feasible when software and clinical process were contextually tailored to each practice environment. If brief lung cancer pre-screening forms are to be implemented, practice contextual factors such as technological capabilities/available software and day-to-day practice functioning would need to be considered.

The current study provides insight into the potential future implementation of electronic lung cancer pre-screening forms in primary care. It is important that research continue to be conducted on potential screening processes in primary care to anticipate and remedy potential issues before a LDCT screening program is widely available.

## Abbreviations

eCHAT: Electronic case-finding and help assessment tool
e-form: Electronic form
EMR: Electronic medical record
LDCT: Low-dose computed tomography
PCPs: Primary care physicians
p-form: Paper form
y: years old
